# Role of ClpP in the Biogenesis and Degradation of RuBisCO and ATP Synthase in *Chlamydomonas reinhardtii*

**DOI:** 10.3390/plants8070191

**Published:** 2019-06-26

**Authors:** Wojciech Majeran, Katia Wostrikoff, Francis-André Wollman, Olivier Vallon

**Affiliations:** 1 Institute of Plant Sciences Paris-Saclay (IPS2), CNRS, Université Paris-Diderot, Université Paris-Sud, INRA, Université Evry, Université Paris-Saclay, Rue de Noetzlin, 91190 Gif-sur-Yvette, France; 2 UMR7141 CNRS/Sorbonne Université, Institut de Biologie Physico-Chimique, 13 rue Pierre et Marie Curie, 75005 Paris, France

**Keywords:** RuBisCO, ClpP, proteolysis *Chlamydomonas*, chloroplast

## Abstract

Ribulose 1,5-bisphosphate carboxylase/oxygenase (RuBisCO) associates a chloroplast- and a nucleus-encoded subunit (LSU and SSU). It constitutes the major entry point of inorganic carbon into the biosphere as it catalyzes photosynthetic CO_2_ fixation. Its abundance and richness in sulfur-containing amino acids make it a prime source of N and S during nutrient starvation, when photosynthesis is downregulated and a high RuBisCO level is no longer needed. Here we show that translational attenuation of ClpP1 in the green alga *Chlamydomonas reinhardtii* results in retarded degradation of RuBisCO during S- and N-starvation, suggesting that the Clp protease is a major effector of RubisCO degradation in these conditions. Furthermore, we show that ClpP cannot be attenuated in the context of *rbcL* point mutations that prevent LSU folding. The mutant LSU remains in interaction with the chloroplast chaperonin complex. We propose that degradation of the mutant LSU by the Clp protease is necessary to prevent poisoning of the chaperonin. In the total absence of LSU, attenuation of ClpP leads to a dramatic stabilization of unassembled SSU, indicating that Clp is responsible for its degradation. In contrast, attenuation of ClpP in the absence of SSU does not lead to overaccumulation of LSU, whose translation is controlled by assembly. Altogether, these results point to RuBisCO degradation as one of the major house-keeping functions of the essential Clp protease. In addition, we show that non-assembled subunits of the ATP synthase are also stabilized when ClpP is attenuated. In the case of the *atpA-FUD16* mutation, this can even allow the assembly of a small amount of CF1, which partially restores phototrophy.

## 1. Introduction

Ribulose-1,5-bisphosphate carboxylase/oxygenase (RuBisCO, EC 4.1.1.39) catalyzes the fixation of CO_2_, a central reaction of the Calvin–Benson cycle. This key photosynthetic enzyme constitutes the main entry point of CO_2_ into the biosphere. RuBisCO enzymes belong to three classes [1]. Forms I and II are present in photosynthetic organisms, while form III is found in archaea, where it performs non photosynthetic functions. The most abundant type of RuBisCO complex belongs to class I and is the one present in cyanobacteria, green algae, and plants. It consists of a ~550 kDa complex formed from the assembly of eight large (LSU) and eight small (SSU) subunits, respectively, encoded by a chloroplast localized *rbcL* and by a small family of nuclear *RBCS* genes (for a review [2]). RuBisCO active sites are formed by adjacent LSU subunits thus making the antiparallel LSU dimer the functional building block. SSU functions to stabilize the complex and may modulate the affinity of substrates. 

RuBisCO appears as a rather inefficient enzyme due to its slow kinetics and low CO_2_/O_2_ specificity [3]. The competing oxygenase activity leads to the synthesis of 2-phosphoglycolate subsequently detoxified in the photorespiratory pathway, and in microalgae by excretion from the cell [4]. Due to its low efficiency, RuBisCO accumulates to high levels in chloroplasts (~60% and ~40% of the chloroplast and total cell protein, respectively). Although these estimations vary between C3 and C4 plants and algae, RuBisCO holds the record of the most abundant protein on Earth [5]. In spite of the presence of an active carbon concentration mechanism, this is also true in the green alga *C. reinhardtii*, where RuBisCO is one of the top 10 most abundant proteins, above all other Calvin cycle enzymes and photosystem II (PSII) subunits [6]. In *Chlamydomonas* and many algae, RuBisCO is mainly localized in a chloroplast subcompartment called the pyrenoid, essential for inorganic carbon concentration mechanisms [7]. SSU has been shown to be essential to form the RuBisCO network-like organization within the pyrenoid, in which the EPYC1 protein functions as a molecular linker [8].

RuBisCO biogenesis is a complex multistep pathway [9,10]. While SSU subunits fold spontaneously after the import to the chloroplast, LSU subunits emerging from the ribosome require the Cpn60/20/10 chaperonin to reach an assembly-competent conformation, as recently reviewed in [11]. Following ATP-dependent release from the chaperonin complex, LSU enters successive assembly intermediates until forming the final holoenzyme LSU_8_/SSU_8_ [12]. This process requires a diverse set of chaperone proteins. These include BSD2, a small DNA-J domain protein that affects C4 bundle sheath differentiation and is essential for RuBisCO assembly in higher plants [13,14], the RuBisCO Accumulation Factors RAF1 and RAF2, first identified in maize [15], and the RBCX chaperone first identified in cyanobacteria [16]. RBCX homologs have been subsequently characterized in *A. thaliana* [17], and conversely, a homolog of RAF1 is present in cyanobacteria [18]. Reconstitution experiments indicated that RAF1 and RBCX, although differing in their site of interaction with LSU, would act in a similar way to stabilize the L2 dimer, opening the way to the subsequent assembly of the complex [19]. BSD2, on the other hand, appears to be specific of land plants. Coexpression of the four above-mentioned auxiliary factors together with the chloroplast chaperonin allows recombinant production of RuBisCO in *E. coli* [20] suggesting that no other chaperone is required for the biogenesis of the holoenzyme. Numerous post-translational modifications have been described on both subunits, concurring to RuBisCO stability and/or activity [2]. Once fully assembled, RuBisCO catalytic activity is maintained by RuBisCO activase [21]. 

RuBisCO biogenesis obeys the so-called “concerted accumulation” rule [22], as the subunits accumulate significantly only if they are assembled. SSU imported in excess is degraded by an unknown protease [23], while LSU production is controlled via inhibition of *rbcL* translation, both in algae [24] and plants [14,25,26]. In addition, LSU molecules unable to reach a productive conformation through interaction with the chaperonin are released to be degraded by proteases [23,27,28]. The chloroplast protease network is therefore implicated in RuBisCO assembly and quality control, but it remains to be established which enzymes are involved.

The chloroplast harbors a large repertoire of proteases [29,30], among which the stromal and thylakoid associated Deg proteases, the thylakoid FtsH protease, the intramembrane rhomboid protease, the thylakoid-bound SppA protease, the Egy1 protease and the Clp protease are the best studied. Here, we focus on Clp, which occupies a central place in this protease network in algae and plants (for review see [31]) and is essential for cell viability in *C. reinhardtii* [32]. It is essentially stromal, like RuBisCO, but a small fraction remains associated with the membrane upon purification [33]. Like its bacterial ancestor, it is composed of two subcomplexes. A hexameric ClpC chaperone, together with accessory proteins, mediates substrate recognition, unfolding and feeding into the proteolytic chamber of the other subcomplex, the ClpP peptidase. The ClpP core is tetradecameric, formed by the association of two heptameric rings. One ring associates the sole chloroplast-encoded subunit, ClpP1, with the nucleus-encoded proteolytically inactive ClpR1-3 subunits, while the other is made up of the active ClpP3-6 [33,34,35,36]. The *clpP1* gene is essential, at least in photosynthetic plastids [37], but its expression can be downregulated in *C. reinhardtii* by mutation of its start codon to AUU [38]. The *clpP1-AUU* mutation causes a ~70% reduction of ClpP accumulation. No growth defect is observed, but the degradation of cytochrome *b*_6_*f* during N-starvation is retarded [38] as well as that of PSII in high light-treated ATP-synthase mutants [39]. Total loss of ClpP has been achieved by conditional repression of *clpP1* [40]. It triggers autophagy and a chloroplast unfolded-protein response, leading to a signaling cascade resulting in an increase in the expression of many proteases, chaperones, and stress-related proteins. To continue our exploration of potential Clp substrates in *Chlamydomonas*, we, therefore, chose the milder *clpP1-AUU* mutation. 

In vascular plants, the massively accumulated RuBisCO constitutes the main intracellular reservoir of nitrogen in green organs, and it can be mobilized through proteolysis during senescence or nitrogen deprivation [41,42]. However, the degradation mechanisms remain unclear, in particular, the balance between stromal proteases and chlorophagy [43]. The physiological responses to N- and S-starvation show interesting similarities in plants [44], as well as in algae [45,46,47]. Among the common responses are growth arrest, oxidative stress and a reorientation of carbon metabolism. Reduced accumulation of RuBisCO has been observed in S-limited conditions in green algae such as *Dunaliella salina* [48] and in *Lemna* [49]. In S-starved anaerobic *Chlamydomonas* cultures, the loss of RuBisCO is extensive [50], but in aerobic conditions, the major response was the degradation of cytochrome *b*_6_*f*, accompanied in high light by that of photosystem II (PSII) [51]. In addition, S-starvation induces the degradation of chloroplast ribosomal proteins [52] and the production of H_2_ as an alternative electron sink to prevent ROS production [53,54]. S-starvation largely impacts the expression of genes implicated in metabolism, protein homeostasis, and oxidative stress response [55]. In *C. reinhardtii*, N-starvation has been extensively studied. It induces an arrest in cell division, and the decoupling of carbon and nitrogen assimilation leads to the accumulation of starch and lipid bodies [56,57]. It also induces gametogenesis [58]. Photosynthetic electron transport is inactivated, involving ClpP-dependent degradation of the cytochrome *b*_6_*f* complex [38,59]. *RBCS* mRNA accumulation is reduced [60], and the RuBisCO protein is degraded, a process that largely depends on the Clp protease [47,61]. 

To further explore the involvement of ClpP protease in RuBisCO biogenesis and degradation, we analyzed the influence of the *clpP1-AUU* mutation in physiological conditions or in genetic backgrounds that activate RuBisCO degradation. These include (i) nutritional sulfur (S-) or nitrogen (N-) starvation that induce massive degradation of the holoenzyme, (ii) RBCS deletion mutants in which LSU is synthesized at a reduced rate and (iii) RbcL mutations leading to rapid degradation of unassembled LSU. We show that RuBisCO degradation is strongly delayed in the context of *clpP1-AUU* mutation, suggesting a major role of the Clp protease in this process. In *rbcL* mutants, we show that LSU remains associated with the Cpn60/Cpn20 chaperonin. In these strains, attenuation of ClpP appears to lead to a non-viable phenotype. Finally, we show that Clp is involved in the degradation of SSU in the absence of LSU. Altogether, these results indicate that RuBisCO degradation is a major house-keeping function of the Clp protease in the chloroplast. In addition, we show that non-assembled subunits of the ATP synthase are also stabilized when ClpP is attenuated. In the case of the *atpA-FUD16* mutation, this can even allow the assembly of a small amount of CF1, which partially restores phototrophy.

## 2. Results

### 2.1. The LSU Assembly Pathway and its Perturbations in RuBisCO Mutants

**Figure 1:**
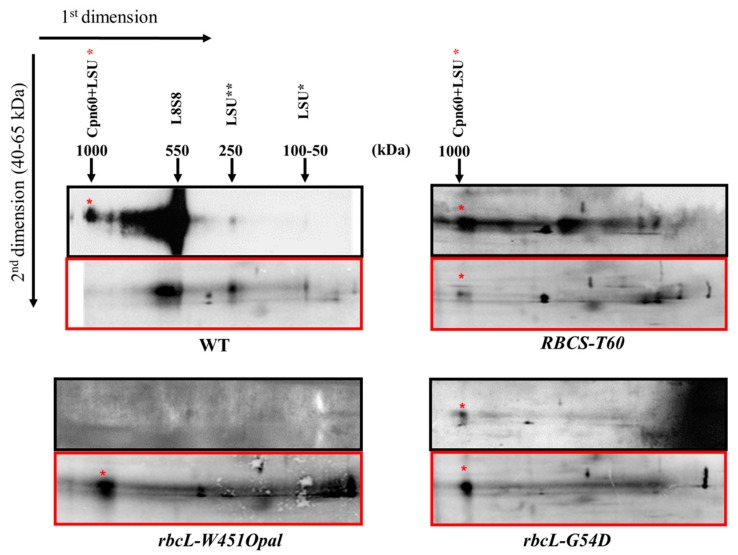
Synthesis and accumulation of LSU oligomeric forms in WT, *rbcL*-G54D, *rbcL*-*W451Opal and RBCS-T60*. Chloroplast-encoded proteins were labeled with ^35^SO_4_^2−^ during 7.5 min in the presence of cycloheximide to block cytosolic translation and separated by 2D-CN-PAGE (4–18% gradient). The accumulation of LSU was followed by western-blot (**top panels**, **in black**) and neosynthesized proteins were detected by autoradiography (**bottom pannels**, **in red**). The mutant panels were overexposed. Only the 40–65 kDa region of the second dimension gel is shown. The estimated molecular weight and nature of the observed complexes are indicated. The red star marks the large subunits (LSU)-chaperonin complex. Black stars indicate putative LSU assembly intermediates.

The *C. reinhardtii rbcL*-G54D (31-4E) and *rbcL*-W451Opal (18-5B) mutants have been characterized as RuBisCO-less strains in which an unstable LSU fails to assemble and is degraded [27,62,63]. To determine the oligomeric state of LSU complexes that are targeted to degradation, we first analyzed RuBisCO assembly using 2D-CN-PAGE separation of soluble proteins. Assembly intermediates were identified by autoradiography after a short ^35^SO_4_^2−^ pulse-labeling, and by western-blotting with an LSU antibody, using the same nitrocellulose filter (Figure 1). The antibody detected LSU mostly as fully assembled RuBisCO holoenzyme (L8S8, ~550 kDa) and as a complex of ~1 MDa (red asterisk) corresponding to the transient association with the chaperonin complex, formed of Cpn60 and Cpn10/Cpn20 subunits [64,65]. Additional faint spots at ~250 kDa and ~100–50 kDa could correspond to assembly intermediates, as they appear stronger in the autoradiogram and become fainter after a 15 min chase (Figure S1C,D). In the mutants, the LSU signals were much fainter than in WT and had to be overexposed (Figure 1). In the *rbcL*-W451Opal strain, LSU could not be detected by western blotting, but it appeared in autoradiograms, exclusively in the 1 MDa complex with the chaperonin. The signals were slightly stronger in the *rbcL*-G54D mutant, but again only the complex with chaperonin could be reliably detected. This may suggest that in both mutants the failure of LSU to fold properly stabilizes its interaction with the chaperonin or that only LSU associated with chaperonins are protected from degradation. However, the low level of accumulation of the mutant proteins indicates that they are eventually delivered to the degradation machinery, either by direct transfer to a protease or after release into the stroma. 

We also analyzed *RBCS-T60*, a mutant that lacks both *RBCS* genes [24]. In this strain, LSU incorporated very little radioactivity, due to the so called control by epistasy of synthesis (CES) process [24] (and results not shown). Here also, the complex with chaperonin could be detected by autoradiography in 2D-CN-PAGE. Western blotting, in addition, showed diffuse bands that may also correspond to assembly intermediates. 

### 2.2. Double Mutants Combining Reduced ClpP Accumulation and Mutant LSU are not Viable

To analyze the role of ClpP in the degradation of the mutant forms of LSU, we attempted to introduce the *clpP1-AUU* mutation in *rbcL*-G54D and *rbcL*-W451Opal strains (Table 1). Chloroplast transformation was carried out by particle bombardment with a plasmid carrying the AUG→AUU mutation at the translation initiation codon of *clpP1*, linked to the *aadA* spectinomycin resistance cassette [38] (Figure S2A). Selection on spectinomycin allowed recovery of resistant transformants having integrated the *aadA* cassette and the closely linked *clpP1-AUU* mutation in their chloroplast DNA. As is usual, due to the presence of ~80 copies of the chloroplast chromosome, transformants were initially heteroplasmic, i.e., carried both mutated and WT alleles of *clpP1*. In the WT genetic background, three to four rounds of sub-cloning on selective media are sufficient to reach homoplasmy, i.e., elimination of the WT *clpP1* allele [38]. In contrast, despite our repeated attempts, the *clpP1-AUU* mutation could never be brought to homoplasmy in strains carrying *rbcL*-G54D or *rbcL*-W451Opal. Transformants were analyzed by PCR for the presence of the *aadA* cassette and of the *Pvu* I restriction site that marks the *clpP1-AUU* mutation [38]. Upon successive subcloning rounds on increasing antibiotics concentration, we observed a progressive loss of the *clpP1-AUU* mutation, while the *aadA* cassette was retained (Figure S2B for *rbcL*-W451Opal, a similar result was obtained for *rbcL*-G54D). Eventually, after 12 rounds of subcloning, homoplasmy for the *aadA* marker was obtained in some strains. However, the *Pvu*I site was lost in all the homoplasmic strains, and thus presumably also the *clpP1-AUU* mutation. This was confirmed by the normal ClpP1 accumulation level in these strains (Figure S2C). Subcloning of the transformants on antibiotics thus appears to select for a recombination event with a WT chromosome in the 376 bp between *aadA* and the *clpP1-AUU* mutation, followed by homoplasmization of the former and loss of the latter. Similar results were observed for the two mutants, in three independent transformants each. These results suggest that the double mutants *clpP1-AUU rbcL*-G54D and *clpP1-AUU rbcL*-W451Opal are not viable. 

### 2.3. Effect of ClpP Attenuation in the Absence of LSU or SSU

**Figure 2:**
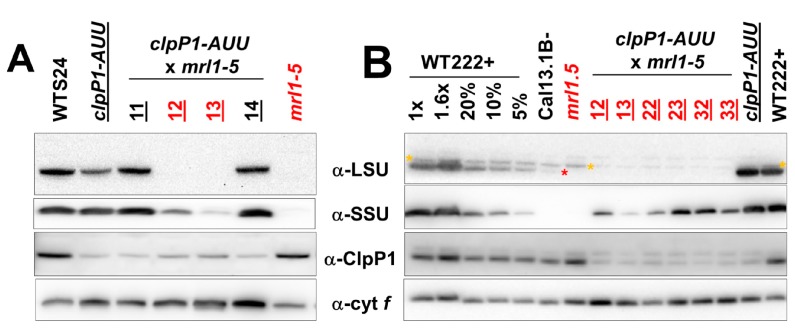
**Effect of ClpP attenuation on the accumulation of SSU in the absence of LSU.** (**A**) Immunoblots showing the steady-state accumulation levels of LSU, SSU, ClpP1 (LMW form) and cytochrome *f* as a loading control, in a cross between a *clpP1-AUU* mt+ strain (ATTK1) and an *mrl1-5* mt- strain. Spectinomycin-resistant strains are underlined, which includes all progeny clones because of uniparental inheritance of the *aadA* cassette. Strains carrying *mrl1-5* (scored as non-phototrophic) are in red. In (**B**), a dilution series of WT proteins mixed with Rubisco-less extracts from the *mrl1-5* mutant is shown for quantification of Rubisco subunit accumulation. Note in the anti-LSU immunoblot in (**B**) the low amounts of LSU detectable in *CAL13* (red *, absent in the *mrl1-5* strains), distinct from an artefactual band marked by an orange *, resulting from the previous reaction of the membrane by the anti-Clp antibody shown in panel α-ClpP1. Samples 12 and 13 in panels A and B are technical replicates.

In contrast, the *clpP1-AUU* mutation could be easily combined by sexual crossing (yielding in *C.reinhardti* an uniparental inheritance of the chloroplast genome containing the *clpP1-AUU* mutation) with a nuclear mutation totally abolishing accumulation of the *rbcL* mRNA (Figure 2), making it clear that in the experiments above, it was not the absence of functional LSU but the presence of a mutated form that prevented homoplasmization of *clpP1-AUU*. The LSU-less strain we chose here was *mrl1-5*, a mutant completely lacking MRL1, the pentatricorepeat protein (PPR) protein that is necessary for stabilization of the *rbcL* mRNA [66,67]. The cross to *clpP1-AUU* did not show abnormal lethality in the progeny, and we readily obtained *clpP1-AUU mrl1-5* recombinants (identified by the total absence of LSU and marked in red in Figure 2). Note that all progeny inherit ClpP attenuation, because the *clpP1-AUU* mutation was provided through the plus mating type parent (strain ATTK1). Western blot analysis revealed that ClpP attenuation partially restored the accumulation of unassembled SSU. In three tetrads analyzed, the *clpP1-AUU mrl1-5* progeny showed levels of SSU accumulation way above the tiny traces observable in the *mrl1-5* parent. It is interesting to note that the degree of restoration of SSU accumulation varied across progeny clones, between 5% and 50% as shown in panel B, even though LSU remained completely undetectable in all clones. This may result from sampling at slightly different stages of growth, as the accumulation of the stabilized subunits in ClpP-attenuated strains declines rapidly as cultures leave the exponential phase [38].

**Figure 3:**
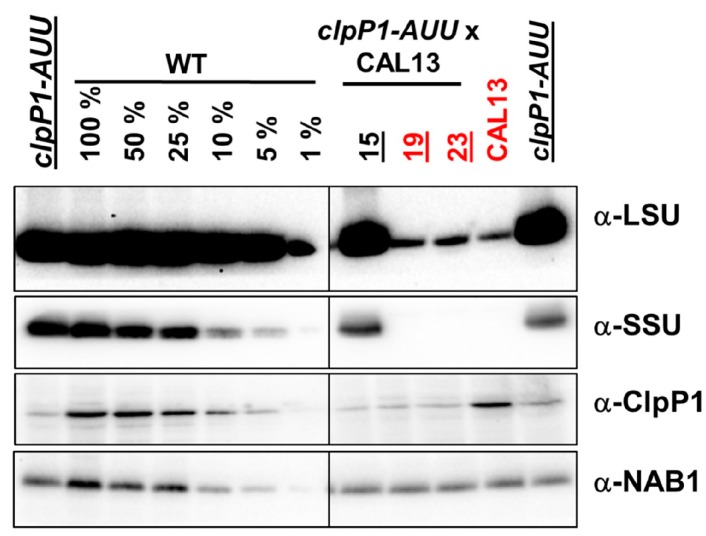
**Effect of ClpP attenuation on the accumulation of LSU in the absence of SSU.** Immunoblots showing the steady-state accumulation levels of LSU, SSU, ClpP1, and the cytosolic protein NAB1, as a loading control. A dilution series of the WT sample is shown on the left, the parents of the cross (ATTK1 mt+ and Cal13 mt-) are shown on the right, with three random progeny clones. Strains carrying the *aadA* cassette (SpecR) are underlined, those carrying the *RBCS* deletion (non-phototrophic, no SSU) are in red. The LSU immunoblot is overexposed to show the low levels observed in the SSU mutants. Note that all progeny clones are SpecR and show the attenuation of the high and low MW forms of ClpP1, whereas only the HMW form is depicted in the figure.

In another cross, aimed at exploring the involvement of ClpP in the degradation of partially assembled but otherwise WT LSU, we combined the *clpP1-AUU* mutation with a deletion of the *RBCS1/2* genes. In our hands, strain *RBCS-T60* did not mate (for reasons probably unlinked to its RBCS deficiency), so we used strain *CAL005_01_13* (hereafter CAL13), obtained from the insertional mutant collection of the Niyogi laboratory [68] where *RBCS1* and *RBCS2* are deleted, together with five neighboring genes, none of which appear relevant to photosynthesis or chloroplast biogenesis. When we examined by western blot the steady-state accumulation levels of Rubisco LSU, we observed no difference between the parent *CAL13* and the *CAL13 clpP1-AUU* progeny (about 1% of WT, Figure 3). This indicates that drastically changing the ClpP level does not affect the stability of the low amounts of LSU synthesized by strains lacking SSU. We have observed that the low level of LSU that accumulates in the absence of its assembly partner is extremely stable (manuscript in preparation). This is generally the case with most CES proteins [69,70,71].

### 2.4. Effect of ClpP Attenuation on the Degradation of Unassembled Subunits in Other Photosynthetic Enzymes

**Figure 4:**
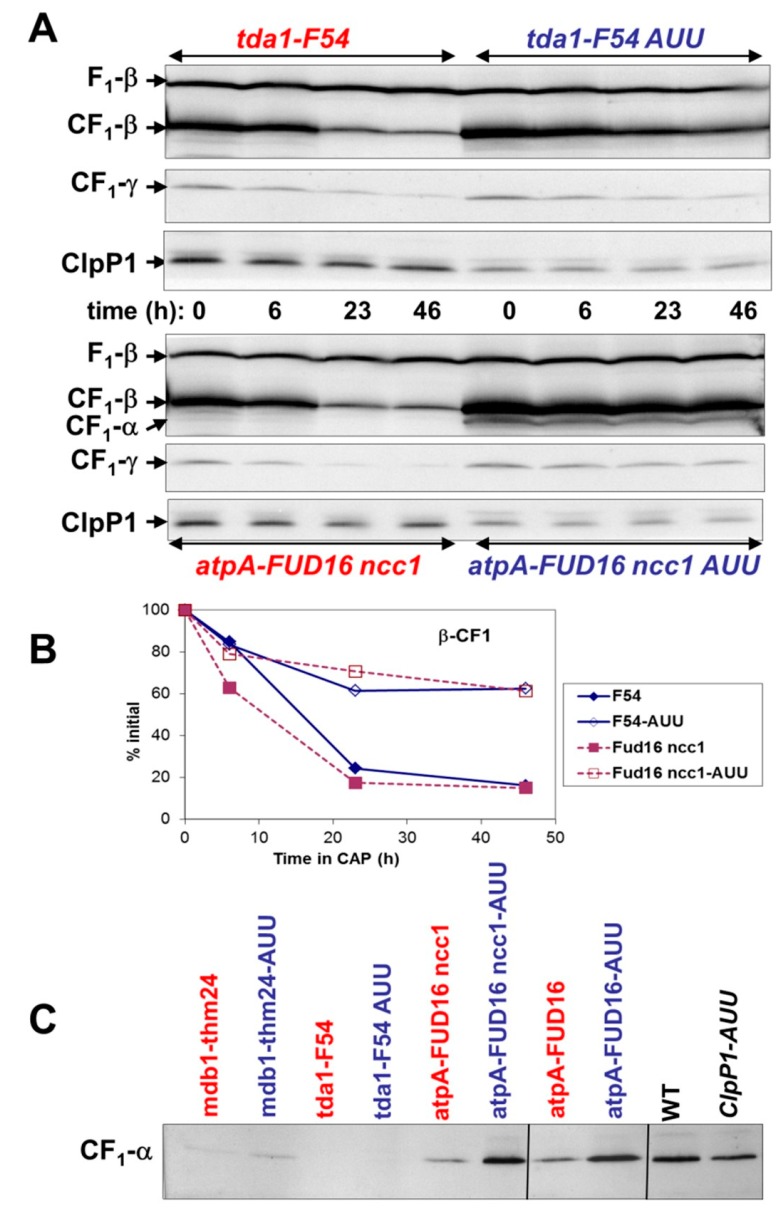
**Effect of ClpP attenuation on the accumulation of unassembled subunits in ATPase mutants.** (**A**): Accumulation of ATPase subunits is shown in *tda1-F54* strains carrying or not the *clpP1-AUU* mutation (upper panels) and in *atpA-FUD16 ncc1* strains carrying or not *clpP1-AUU* (lower panels) after addition of chloramphenicol (time 0). The levels of ClpP1 and CF1 subunits were followed by western blotting with antibodies to β- and α-CF1 (top) γ-CF1 (middle) and ClpP1 (bottom). The β subunit of mitochondrial F1 cross-reacts with the β-CF1 antibody, providing an internal loading control. In ClpP-attenuated strains (open symbols), the degradation is retarded. (**B**): Quantification of the level of β-CF1 as the percentage of the initial value. (**C**) Immunodetection of α-CF1 in soluble fractions obtained by Yeda press treatment of exponentially growing WT and ATPase mutants, carrying (blue) or not (red) the *clpP1-AUU* mutation.

In previous studies, we have shown that the *clpP1-AUU* mutation stabilizes the non-assembled mutant Rieske protein in the *petC-ac21* mutant [38]. To investigate whether ClpP attenuation would similarly stabilize other incompletely assembled photosynthetic enzymes, we have combined ClpP attenuation with various photosynthesis mutations. For example, the *tbc1-F34* mutation prevents translation of CP43, a subunit of the PSII core [72]. In a *tbc1-F34 clpP1-AUU* double mutant, we observed no restoration of the accumulation of the two other integral PSII subunits that we examined, CP47 and PsbH (results not shown). In contrast, when we examined mutations affecting the assembly of the ATPase CF1, which occurs in the stroma, we found a stabilizing effect of ClpP attenuation. The *tda1-F54* mutation has been shown to completely prevent translation of α-CF1, and β-CF1 accumulates in the stroma to about 10% of WT [73]. As expected, the attenuation of ClpP did not restore accumulation of α-CF1 in the *tda1-F54* mutant, but it markedly increased the accumulation of β-CF1 compared to the *tda1-F54* parent (Figure 4C). To directly follow the degradation of the unassembled β subunit, CAP was added to stop production of chloroplast-encoded subunits. In *tda1-F54 clpP1-AUU*, the degradation of β-CF1 was much slower than in *tda1-F54* (Figure 4A,B). This indicates that in the absence of α-CF1, the Clp protease participates in the degradation of the unassembled β-subunit.

In the *mdb1-thm24* mutant background, where no *atpB* mRNA accumulates and β-CF1 is not produced [74], attenuation of ClpP had little if any effect on α-CF1 accumulation (Figure 4C). In the absence of β-CF1, the CES process leads to an inhibition of α-CF1 translation [69], and proteolysis is thus not expected to play a major role in controlling accumulation level as for LSU and other CES subunits. However, attenuation of ClpP did stabilize α-CF1 in strains carrying the *atpA-FUD16* mutation. This mutation changes two residues in α-CF1 (I184N and N186Y), causing it to aggregate in the stroma and form inclusion bodies which also contain β-CF1 [75]. When *atpA-FUD16* was combined with *clpP1-AUU*, the accumulation of α-CF1 in the soluble fraction markedly increased (Figure 4C), but inclusion bodies still formed. To examine the effect of ClpP attenuation in the absence of inclusion bodies, we took advantage of the observation that they do not form when *FUD16* is combined with *ncc1*, a mutation that destabilizes the *atpA* monocistronic mRNA and thus reduces the translation rate of α-CF1 [74,76]. In these conditions, the mutant protein no longer aggregates and instead is rapidly degraded [75]. When ClpP was attenuated in an *atpA-FUD16 ncc1* background, the accumulation of α-CF1 in the soluble fraction dramatically increased (Figure 4C) and the mutant α-CF1 now was detectable in whole cell extracts and extremely stable during CAP treatment (Figure 4A). Note also that β-CF1 is also stabilized in this background (Figure 4A,B and Figure S3). Interestingly, γ-CF1 also appeared stabilized in *atpA-Fud16 ncc1 clpP1-AUU* (Figure 4A), which led us to ask whether some functional ATPase could be assembled in this strain, in spite of the mutations in α-CF1. When we analyzed the assembly state of CF1 by 1D-CN-PAGE (Figure S3), we found that a faint band containing both α- and β-CF1could be observed in the soluble fraction of *Fud16 ncc1AUU*, and to a lower extent of *Fud16 AUU*, exactly where CF1 runs in the WT. These results were confirmed by 2D-BN-SDS-PAGE (not shown). Consistent with these findings, we could observe (Figure S4A) a very slow photosynthetic growth of *Fud16 ncc1AUU*, in minimum media liquid cultures and under moderate light. No phototrophy was observed in spot tests on agar plates. The final proof of the existence of a functional thylakoid-bound ATPase in this strain came from measurements of the decay of the flash-induced electrochromic shift (Figure S4B). The decline in the signal, which largely reflects the proton-channel activity of the ATP synthase, was rapid in the WT and slow in the ATPase mutants, as shown previously [73]. In the *Fud16 ncc1* background, ClpP attenuation led to a partial restoration of the decay rate. The ATPase activity observed in *Fud16* was very low, as in the original study, and was not stimulated by ClpP attenuation, consistent with the fact that neither strain was amenable to phototrophic growth. 

### 2.5. RuBisCO Degradation during Sulfur and Nitrogen Starvation is ClpP Dependent

Because RuBisCO is known to be affected by various nutritional stresses, we asked whether ClpP could be involved in the degradation of the fully assembled enzyme, and not just the unassembled subunits. We subjected *C. reinhardtii* strains with normal or reduced ClpP levels to sulfur (S-) or nitrogen (N-) starvation and monitored the accumulation of RuBisCO and its rates of synthesis, assembly, and degradation. When cells initially grown on TAP were transferred to sulfur-depleted medium (TAP-S), measurements of fluorescence induction kinetics showed a reduction in variable fluorescence, a measure of PSII activity. Only limited differences were observed between WT and ClpP-attenuated strains (Figure S5A), suggesting that the degradation of PSII during S-starvation, already described in [51], is largely independent of ClpP levels. However, the loss of overall efficiency of photosynthetic electron transfer in vivo, for which we took ΦPSII (Fm-Fs/Fm) as a proxy, was less rapid in *clpP1-AUU* strains until the 48h time point, after which it collapsed (Figure S5B). Recovery experiments, performed by re-introducing sulfur in the growth medium, showed a fast (<20 h) recovery of the initial electron transfer efficiency, suggesting a rapid re-build of the photosynthetic apparatus. 

**Figure 5:**
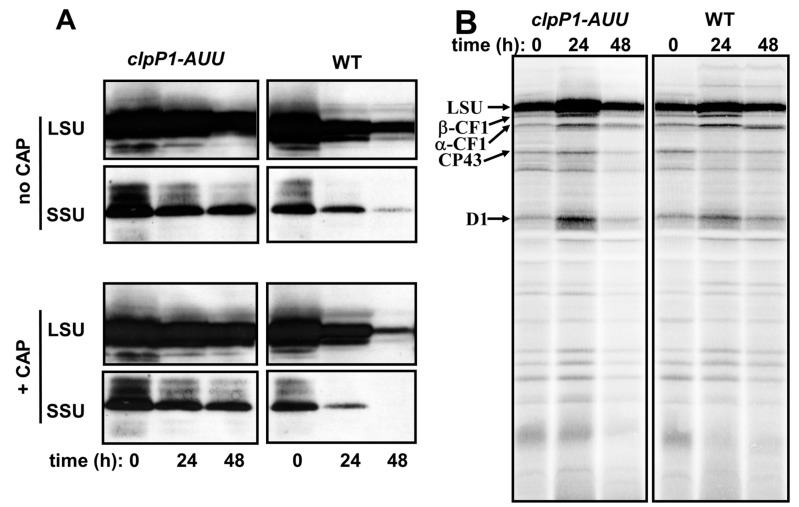
**RuBisCO degradation during S-starvation.** (**A**) Cells of the *clpP1-AUU* and WT strains were placed in S-free medium in the absence (top) or presence (bottom) of chloramphenicol (CAP). Accumulation of LSU and SSU was analyzed by western-blot with the anti-RuBisCO antibody after 0 h, 24 h and 48 h of starvation. (**B**) Chloroplast-encoded proteins from *clpP1-AUU1* and WT were labeled with ^35^SO_4_^2−^ in presence of cycloheximide, before or after 24 h or 48 h S-starvation. The position of LSU and a few other chloroplast proteins are indicated by arrows. Rather than enhanced chloroplast translation, the overall higher ^35^S incorporation observed at 24 h probably reflects an increase in the efficiency of sulfate metabolization in response to starvation [77].

The observation above suggests that a component of the photosynthetic apparatus downstream of PSII is degraded in response to S-depletion, possibly in a ClpP-dependent manner. When RuBisCO accumulation was monitored by western-blot (Figure 5A), we found that after 48 h of starvation, the LSU and SSU signals dropped down in the WT to respectively 20% and 3% of their initial levels. However, in *clpP1-AUU* mutants the decline of RuBisCO was dramatically delayed, leaving LSU and SSU signals at respectively ~80% and ~50% of their initial value. The apparent larger loss of SSU is probably due to a non-linear response of the antibody signal rather than to a difference in degradation kinetics, because LSU oligomers in 2D-CN-PAGE appeared almost exclusively in the L8S8 form, even in pulse-chase experiments (see Supplementary Figure S1 for *clpP1-AUU*). The rate of LSU translation appeared to be similar in the ClpP-attenuated and control strains (Figure S5B), and both appeared to lose the *rbcL* and *RBCS* mRNA at a similar pace during the first day of starvation (Figure S6A). The *clpP1-AUU* mutation, therefore, appears to act at the post-assembly level, i.e., on the degradation of L8S8. Performing the S-starvation experiment in the presence of Chloramphenicol (CAP) allowed us to directly follow the kinetics of RuBisCO degradation, i.e., without interference from LSU translation (Figure 5A, lower panels). In both strains, CAP slightly accelerated the degradation of both RuBisCO subunits, confirming that in the absence of the drug, some synthesis of RuBisCO still occurred during the course of starvation. Importantly, the contrast between the WT and the *clpP1-AUU* mutant was increased by CAP treatment, when no de novo translation can compensate for RuBisCO degradation. Altogether, the results above indicate that ClpP is a major determinant of RuBisCO degradation during S-starvation. 

**Figure 6:**
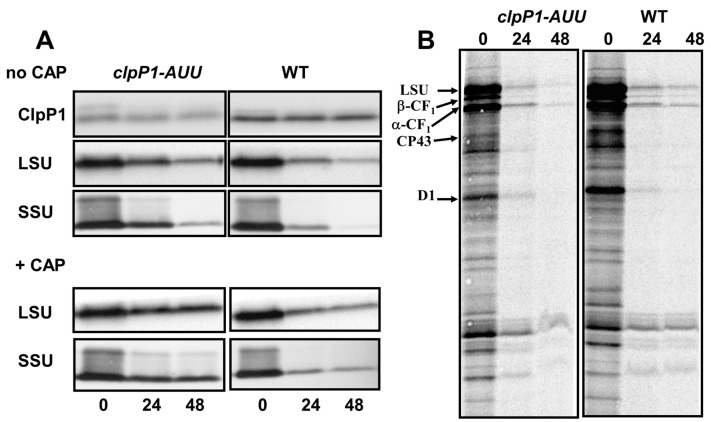
**RuBisCO degradation during N-starvation.** (**A**,**B**) Same as Figure 5, except that starvation was in N-free medium and pulse labeling was performed with ^14^C-acetate. Note the weakness of pulse-labeling signals in N-free medium, probably reflecting weaker incorporation of acetate in these conditions.

N-starvation also led to the degradation of RuBisCO (Figure 6). In the WT, after 48 h in N-free medium, western-blot showed a reduction of LSU and SSU signals, respectively down to 22% and 5% of their initial level. In the *clpP1-AUU1* mutant, the decrease of RuBisCO accumulation was strongly delayed, leaving LSU and SSU, respectively to ~50% and 20% of the initial value after 48 h of starvation (Figure 6A). These figures are similar to those reported by [47]. To further explore the mechanism of this stabilization, a short pulse labeling was performed, here with ^14^C-acetate. It showed no major difference between WT and ClpP-attenuated strains (Figure 6B): In spite of reduced incorporation of ^14^C-acetate in N-starved cells, the autoradiogram shows that LSU was similarly affected as the other chloroplast-encoded proteins. The *rbcL* and *RBCS* mRNA levels (Supplementary Figure S6B) dropped even more rapidly than during S-starvation, but again, similarly in the two strains. Similar to S-starvation, CAP treatment increased the contrast between WT and *clpP1-AUU* strains (Figure 6A, lower panels). Altogether, these results confirm that ClpP is a major determinant of RuBisCO degradation also during N-starvation. 

## 3. Discussion

### 3.1. Role of ClpP in the Degradation of Assembled RuBisCO

Nutrient starvation in *Chlamydomonas* has been extensively studied, not only because it promotes the accumulation of triacylglycerides of potential biotechnological interest, but also because it can inform us on plant stress responses and leaf senescence. One of the common hallmarks of senescence and stress-responses in plants and algae is the inactivation of photosynthesis, often associated with the degradation of chloroplast components, in particular, proteins that then serve as a source of nutrients. The degradation of RuBisCO and other chloroplast proteins in senescing leaves, despite its importance for pre-harvest N remobilization and thus for crop yield, is still incompletely understood [41]. It is believed to occur both inside and outside the chloroplast [78], but the identity of the protease(s) involved is still under debate. A systematic study of protease activities induced during senescence [79] offers a long list of candidates, in particular, papain-like cysteine proteases, but none appears chloroplast-localized. Inside the organelle, a chloroplast metallo-endopeptidase EP1 has been implicated [80], but it has not been identified genetically. In tobacco, a chloroplast-DNA binding aspartyl protease CND41 has been proposed to degrade RuBisCO during senescence induced by N-starvation [81,82]. CND41 was purified from chloroplasts [83], but its in vivo localization was never fully investigated. Its reported pH optimum of 2.5 [81,82] is not compatible with a function in the stroma. Furthermore, the proposed mature N-terminal residue (K121) lies, in an alignment of the 60 closest CND41 homologs that we collected from plant and green algae, within a well-conserved region (Figure S7). This would be highly unusual for a chloroplast imported protein where sequence conservation generally starts after a comparatively short transit peptide but is more fitting with the cleavage of a propeptide, a frequent mechanism for post-translational activation of secreted peptidases.

Finally, TargetP predicts as secreted 33 of the 49 CND41 homologs for which the N-terminal sequence appears reliable, including tobacco CND41 itself. We conclude that CND41 and its orthologs (among which *Chlamydomonas* ASP2) are probably vacuolar rather than stromal proteases. If CND41 participates in RuBisCO degradation, it probably acts in an acidic vacuolar compartment and not in the chloroplast itself. 

This is also probably true of the other proteases that have been implicated in RuBisCO degradation during plant senescence, including the papain-like cysteine protease SAG12 [84]. Interestingly, the latter study showed that mutation of SAG12 led to three-fold induction of AT5G10760, the closest homolog of CND41 in Arabidopsis. After many years of research, autophagy has come to be recognized as the main mechanism for RubisCO degradation in stressed or naturally senescing leaves (for reviews see [43,85]). Small autophagy-dependent RuBisCO-containing bodies can be visualized in the plant vacuole, arising from chloroplast protrusions into the vacuole [86,87]. Autophagy is also responsible for the larger “ATI bodies” and may even degrade entire chloroplasts, a process called chlorophagy. In addition, autophagy-independent processes have been described that also mediate extra-chloroplastic degradation of chloroplast fragments: chloroplast vesiculation containing vesicles (CCV) lead to degradation in the vacuole, while Senescence-associated vacuoles (SAV), containing SAG12, function as lytic compartments by themselves [43].

In *Chlamydomonas*, N-starvation activates the autophagy pathway [88,89], as does inhibition of chloroplast fatty-acid synthesis [90]. However, even though chloroplast functions were deeply affected in these conditions, direct evidence for autophagic destruction of chloroplast material has not been reported. We also note that some of the pathways described in plants probably have no counterpart in *Chlamydomonas*: ATI1 (AT2G45980) which is responsible for the ATI bodies, is absent from algae, while chloroplast vesiculation (AT2G25625), the gene responsible for the formation of CCVs, has homologs only in angiosperms. The *Chlamydomonas* genome contains papain-like cysteine proteases, but none appear closely related to SAG12. This alga thus appears to be a good model to explore the intra-chloroplastic degradation of RuBisCO. 

Until now, ClpP is the only chloroplast localized protease that has been convincingly linked with the degradation of RuBisCO, and this only in N-starved *Chlamydomonas* [47,61]. The more recent study also showed that FtsH was not involved in RuBisCO degradation. Here, we confirm that ClpP attenuation by the *clpP1-AUU* mutation retards RuBisCO loss in N-starved cultures. We provide further evidence that this is indeed due to a slowing down of its degradation, rather than to compensating mechanisms operating at the transcriptional, translational or assembly level. Combined with the observation that ClpP attenuation also leads to stabilization of cytochrome *b*_6_*f* during N-starvation [38], this indicates that proteolytic mechanisms play a major role in the reorientation of chloroplast metabolism from an energy-storage to an energy-dissipation mode during N-starvation [47]. However, while Clp apparently has a direct impact on the stromal RuBisCO, the thylakoidal cytochrome *b*_6_*f* appeared to be degraded mostly by FtsH [47]. The observed effect of ClpP attenuation on the degradation of cytochrome *b*_6_*f* was therefore re-interpreted as indirect, reflecting a shift in FtsH activity caused by the crippling of the major stromal protease. Chloroplast proteases may have distinct target specificities, but the effect of their impairment in vivo must always be considered in the framework of the integrated network that they form together with their substrates and modulators. 

In higher plants, an indirect hint that Clp may be involved in the senescence program is the induction of Erd1 (ClpD) and of the catalytic subunits ClpP3 and ClpP5 during natural senescence in Arabidopsis [91]. These observations are confirmed by the GFP-based expression atlas at TAIR (https://www.arabidopsis.org/) which in addition reveals ClpR1 as induced during senescence. However, the proteomic analysis of mutants in various ClpP, ClpR, ClpS or ClpC genes (summarized in [92]) does not place RuBisCO in the list of potential targets. In contrast, impairing Clp function had a negative effect on RuBisCO accumulation, i.e., the mutants overall had less RuBisCO than the WT. This was related to the pale-green and generally stressed phenotype of the plants and, like most of the responses observed, interpreted as a secondary effect of a general defect in chloroplast protein homeostasis. Because of the developmental retardation in the mutants, these studies had to be calibrated on leaf stage and they were not pursued through senescence. The question of whether the Clp protease contributes to RuBisCO degradation during senescence of plant leaves, therefore, remains open.

The sulfur-deprivation response of green algae shares many characteristics with that to nitrogen-starvation [93]. Both entail growth arrest, increase in cell size and carotenoid content, overaccumulation of neutral lipids and carbohydrates and loss of chlorophyll and proteins (see [94] and [95] for comparative studies in *Chlamydomonas* and *Dunaliella*, respectively). Because storage of fixed C in lipid droplets and starch granules saturates progressively, it is not surprising that prolonged starvation also leads to the inactivation of photosynthesis, balancing the inactivation of net amino-acid production and cessation of growth. Only limited amounts of readily-mobilized forms exist for both N and S in a growing cell, so besides induction of scavenging systems aimed at restoring nutrient flux from the environment, amino-acids are recycled from the protein stores. When starvation is performed in closed photobioreactors, RuBisCO is the primary target of degradation, followed by photosystem II (PSII) [50,96]. Anoxy is established, allowing photo-production of H_2_. PSII inactivation has also been observed in open flasks [51], as well as the degradation of chloroplast ribosomal proteins [52]. In rice, S-starvation also leads to the degradation of RuBisCO [44].

Here, we show that the degradation of RuBisCO in S-starved *Chlamydomonas* is also dependent on ClpP. The effect of ClpP attenuation is even stronger than during N-starvation, leaving little doubt that RuBisCO indeed is a substrate for the Clp protease in these conditions. Together, the LSU and SSU sequences harbor a large proportion of sulfur-containing residues: 76/660, i.e., 11.5%, compared to 6.1% for *Chlamydomonas* proteins in general. This makes RuBisCO an excellent intracellular source of S, especially when its overaccumulation is no longer needed to sustain phototrophic growth. Another situation where RuBisCO and ClpP may interact is in the shift between low and high CO_2_ in high light. In these conditions, the *clpP1-AUU* mutation, in an otherwise WT context, leads to massive cell death [38]. The inability of the mutant to manage the shift may be due to the impaired degradation of RuBisCO or associated proteins, at a time when the pyrenoid is massively reorganized [8].

What is the trigger signal converting RuBisCO into a substrate for Clp? One unlikely possibility is that new effectors are expressed during stress that would specifically recognize RuBisCO and unfold it or otherwise target it to the degradation machinery. Covalent modifications of the enzyme are more likely causes of the destabilization. Non-enzymatic breakage within the LSU backbone at Gly-329 can be triggered by Fe^2+^ ions in oxidative conditions, and it has been proposed as a signal for RuBisCO degradation [97,98]. The damaged L8S8 may then become a target for the Clp protease. Alternatively, sensing of RuBisCO redox status through the redox state of Cys-449 and Cys-459 has been implicated in its sensitivity to proteases in vitro [99]. A similar redox-based mechanism was proposed to regulate RuBisCO association with chloroplast membranes [100]. Irreversible post-translational modifications of LSU or SSU could also act as a “tag” to target the enzyme towards degradation, either because they are directly recognized by the protease or because they lead to partial disassembly. In *Chlamydomonas*, nitric oxide (NO) acts as a signal for the degradation of cytochrome *b*_6_*f* during N-starvation [46,47], and one of the proposed mechanisms is the nitrosylation of one of its subunits. A similar mechanism may be at play for RuBisCO during N- or S-starvation. Proteomic studies aimed at detecting covalent post-translational modifications of chloroplast proteins would certainly help clarify this point. 

Note that the degradation of a multi-subunit complex, be it membrane-embedded or soluble and be it carried out by Clp or FtsH, poses a specific challenge to the protease. If these hypothetical modifications were sufficiently disruptive to cause the individual subunits to fall apart as a prerequisite for their being recognized as substrates by the protease, then the attenuation of the protease should not markedly stabilize them: The detached subunits would always end up being degraded faster than they could ever reassociate with the other detached subunits. We must, therefore, imagine that the chaperone moiety of the protease (the ClpC/D complex or the AAA+-domains of the hexameric FtsH) recognizes the target enzyme in the assembled state. It would then destabilize the interaction between subunits, so that it can feed them one by one into its unfolding cavity and then into the proteolytic chamber of the peptidase moiety. In the case of Clp, ClpC is usually considered more abundant that the ClpPR complex, so the form that is active on RuBisCO is probably the complete ClpC/ClpPR holoenzyme, whose accumulation level is directly limited by the *clpP1-AUU* mutation. In our 2D-gels, no partially disassembled forms of RuBisCO could be observed during S-starvation, but they are probably transiently formed during the degradation process.

### 3.2. ClpP Levels Control the Accumulation of Unassembled Subunits of Photosynthetic Complexes.

The SSU branch of the assembly pathway is simpler than the LSU branch. It does not appear to require specific chaperones or assembly factors, except for a possible interaction with RAF2 [9]. SSU transiently interacts with Cpn60/Cpn20 [101], as do other imported proteins. In some cases, binding of the imported protein to the stromal chaperonin seems to follow interaction with HSP70 [102] or precedes it [103]. It has long been recognized that assembly is the limiting factor for SSU stability, as newly imported SSU was rapidly degraded in the absence of LSU [23]. In this work, we show that Clp is probably the protease responsible for this degradation. ClpP attenuation leads to a dramatic stabilization of SSU in the absence of LSU, from barely detectable traces to half of the WT level. RuBisCO being a major protein in the organelle and the biogenetic flux of SSU being unaffected by the absence of LSU, the degradation of SSU in these conditions probably represents a sizeable fraction of the Clp proteolytic burden. Still, the attenuation of ClpP in this context did not lead to noticeable deleterious effects on growth, which suggests that the accumulation of unassembled SSU is easily accommodated by the chloroplast. A mutant Rieske protein can also be stabilized by ClpP attenuation in the *petC-ac21* strain [38]. 

In contrast, the accumulation level of LSU in SSU mutants is not dictated by its degradation, but by its translation rate [24]. An assembly-dependent translational regulation known as “Control by Epistasy of Synthesis”, or CES process [104] limits the translation of key subunits of the photosynthetic complexes (LSU, cytochrome *f*, D1 and CP47, PsaA and PsaC, α- and β-CF1) when their assembly partners are missing [69,70,105,106]. In general, the regulated subunits are the most stable in the complex, which allows them to act as repressors of their own synthesis and at the same time justifies the need for regulation of their production. We were therefore not surprised to observe that ClpP attenuation did not lead to overaccumulation of LSU in the SSU-less mutant CAL13. Due to the negative feedback loop, a stabilization of the still unknown LSU assembly intermediate that mediates CES inhibition would be expected to stimulate the CES mechanism and further inhibit translation of the *rbcL* mRNA. An interesting prediction of this model actually is that if ClpP is involved in the degradation of the repressor assembly intermediate, then introducing the *clpP1-AUU* mutation in a CAL13 background would lead to a further downregulation of the *rbcL* translation rate. Unfortunately, the low level of radiolabel incorporation in CAL13-*clpP1-AUU* strains prevented precise measurement of LSU synthesis.

CF1 is another well-studied complex associating chloroplast- and nucleus-encoded subunits. Its α-, β-, γ- and ε-subunits assemble in the stroma before associating with the membrane-bound CFo moiety to form the thylakoid ATP synthase [107]. At least two feedback loops regulate the translation of α and β [69]. Among the plethora of ATPase mutants available in *Chlamydomonas*, we started with *tda1-F54* and *mdb1-thm24* as they totally lack respectively α and β. In the absence of α, attenuation of ClpP leads to a marked stabilization of unassembled (Figure 4), which suggests that the Clp protease is a major contributor to the degradation of β in the stroma. In the WT, free β drives the system forward by stimulating translational initiation on the *atpA* mRNA [69], so that excess production of β will lead to a compensatory stimulation of α production. The Clp protease here has the potential to moderate this effect, as it can eliminate a fraction of this extra β. We have not seen an overaccumulation of β in the stroma in the *clpP1-AUU* mutant (Figure S3) and no changes in the translation of α (e.g., Figure 4), but this is probably because in a WT context assembly is fast and the small amounts of free β rapidly enter the assembly pathway. However, in conditions where translation of α is reduced and more unassembled β accumulates, a positive effect of ClpP attenuation could be expected.

It is more difficult to determine whether unassembled α is also a substrate for Clp, because the absence of β (or of γ which leads to severe downregulation of β synthesis) immediately limits translation of α. The signal obtained for α in the *mdb1-thm24* mutant (Figure 4C) is extremely faint and it is hard to decide whether it is enhanced by ClpP attenuation. This is why we turned to a mutant version of α encoded by *atpA-FUD16*. Due to point mutations in surface-exposed residues, the protein aggregates to form inclusion bodies, probably because the αβ oligomers form but cannot associate into a CF1 able to accommodate γ [75]. Proteolytic degradation competes with the aggregation pathway: In the *Fud16 ncc1* double mutant where translation of α is depressed due to a reduction in accumulation of the monocistronic *atpA* mRNA, the mutant protein does not aggregate. Our finding that attenuation of ClpP in this genetic background restores accumulation of α indicates that the Clp protease is largely responsible for the degradation of the mutant α-subunit. ClpP attenuation allows it to accumulate in the soluble fraction, partly as small aggregates but probably also in a form that allows productive assembly. A complex of electrophoretic mobility similar to CF1 is formed in the stroma, and our results suggest that it can associate with CFo to form a function enzyme. The observation of a weak capacity for photoautotrophic growth (Supplementary Figure S4A) in itself would not be sufficient proof, as metabolic cooperation between the chloroplast and mitochondrion can in some conditions restore phototrophy even in the absence of chloroplast ATPase [108]. However, the acceleration of the decay rate of the electrochromic signal (Supplementary Figure S4B) clearly points to a partial restoration of ATPase activity. This assay can detect activity levels below those required for phototrophy, as some activity was already reported for FUD16 [75]. Thus only a proper balancing of the biogenesis and proteolysis rates could reveal the phototrophic potential of strains carrying the *FUD16* mutation. Similarly, studies of the *petC-ac21* mutant [38,109] and of Q-cycle mutants [110] have shown that downregulating proteolysis is a powerful tool to identify genetic backgrounds allowing low levels of activity of a photosynthetic enzyme that normally would not allow phototrophy.

### 3.3. ClpP is Necessary for the Proteolytic Disposal of Mutant LSU

Point mutations in *rbcL* have greatly contributed to our understanding of RuBisCO catalysis and assembly [111]. Here, we further explore the consequences of two well-characterized mutations that prevent folding of LSU, G54D, and W451Opal, known to completely prevent the formation of a stable enzyme [27,62,63]. Our 2D-CN-PAGE analysis shows that in both cases, LSU is detectable only in association with the chaperonin complex. Already in 1989, Avni et al. had found that a mutant LSU in tobacco was entirely associated with the chaperonin in the so-called "B-complex" [112]. This is in line with the proposed mechanism for chaperonin action: It accommodates improperly folded proteins in its internal cavity and undergoes cycles of ATP hydrolysis and conformational changes until the protein emerges in its folded state [113]. The mutant LSU never reaches this stage and might, therefore, remain forever inside the chaperonin cavity. Given the high translation rate of *rbcL*, this would pose the threat of irreversibly poisoning the chaperonin. Interestingly, an over-accumulation of the chaperonin was observed in [112], which might very well reflect a regulation of chaperonin biogenesis to compensate for this partial inactivation. Chaperonin function is essential in the chloroplast, probably because of its central role in folding soluble chloroplast-encoded and imported proteins, but also of its more specialized functions such as in forming the FtsZ ring for plastid division [114]. So the survival of *rbcL* mutants must depend on mechanisms liberating the chaperonin from unfoldable substrates. Based on our observation that even the moderate reduction in ClpP imparted by the *clpP1-AUU* mutation is not tolerated in the context of an *rbcL* point mutation, we propose that the Clp protease is responsible for this function. The ClpPR complex might even be capable of performing such a function by itself, with no need for the ClpC/D chaperone, if it associated with the chaperonin, allowing direct transfer of the unfolded substrate into the proteolytic chamber. Whatever the mechanism, the strong synthetic lethality we observed between *clpP1-AUU* and *rbcL* point mutations speaks in favor of a tight coupling between LSU folding and proteolytic quality-control processes. 

## 4. Materials and Methods

### 4.1. Strains and Culture Conditions

We used *C. reinhardtii* wild type strains WT11 *mt+*, WTS24 *mt-* and WT222 *mt+*, all derived from strain 137C. Transformants BI (WT11 transformed with the *aadA* cassette), ATTK1 and ATTK2 (carrying *clpP1-AUU*) have been described [38]. RuBisCO mutants *rbcL*-G54D, *rbcL*-W451Opal and *RBCS-T60* were obtained from R. Spreitzer, while Cal.005.013 [115] was obtained from the Niyogi lab. Cells were grown on Tris-acetate (TAP) medium [116] at 25 °C under continuous illumination (50 µmol.m^−2^.s^−1^ for the photosynthetic, ~7 µmol.m^−2^.s^−1^ for the non-photosynthetic strains). For nitrogen and sulfur starvation, cells grown in Erlenmeyer flasks (200 mL of culture in 500 mL flasks) with orbital shaking (~100 rpm) were collected in exponential phase of growth (~10^6^ cells/mL), washed twice by centrifugation with ¼ of initial culture volume in nitrogen- or sulfur-free media (TAP-N, TAP-S) and resuspended in one volume of TAP-N or TAP-S. Where indicated, chloramphenicol (CAP) was added at 100 µg/mL to block chloroplast translation. For photoautotrophic growth experiments with *Fud16 ncc1AUU* were performed in liquid minimum medium (Figure 4A).

### 4.2. Chloroplast Transformation

RuBisCO mutants were transformed by tungsten particle bombardment [117] with a compressed-air shotgun device built in the laboratory by D. Beal and P. Bennoun. The selection and screening of transformants were performed under very dim light, as described in [38]. Briefly, the presence of the transforming DNA was detected by Polymerase Chain Reaction (PCR). Primers PA1 (59-GCAGAATCTTTGTCTTGATTAGGTG-39) and AA1 (59-CACTGCCTCTAATAAAGTCATCG-39) were used to specifically amplify a 0.91 kb region between *clpP1* and *aadA*. Amplification products were digested by P*vuI* to verify the presence of the restriction site associated with the *clpP1-AUU* mutation. 

### 4.3. Biochemical Analysis and Fluorescence Measurements.

SDS-PAGE was performed on 12 to 18% urea-containing gels [118]. Proteins were electroblotted onto nitrocellulose [119] and immunodetection was performed using ^125^I-Protein A, and the fluorescence signal revealed with a Phosphorimager. For Figure 2 and Figure 3, immunodetection used the ECL system (Amersham). Antibodies to ClpP1, *Chlamydomonas* RuBiscO, land plant LSU and SSU were described previously [38]. A second antibody directed against land plant Rubisco and kindly provided by Spencer Whitney (ANU Canberra, Australia) was used in Figure 2 and Figure 3. Colorless Blue native PAGE (CN-PAGE) were performed accordingly to [120], using 4–18% linear gradient gels. Total soluble protein extracts that were separated on CN-PAGE were prepared as described in [33]. Estimated native molecular weights of observed complexes were calculated in reference to a set of native molecular weight markers. Northern blots were performed according to [74]. Chlorophyll fluorescence induction curves were obtained and analyzed as in [38].

### 4.4. Pulse Labeling and Chase 

Pulse and chase experiments were performed on cells harvested in the mid-exponential growth phase (~2 × 10^6^ cells/mL) or nutrient-starved. For ^35^S-labeling, cells were washed and resuspended at the same density for 30 min in TAP-S medium, then labeled by addition of ^35^SO_4_^−^ (200 μCi) during 7.5 min in presence of cycloheximide (15 μg/L). Cells were disrupted by Yeda-press (100 bar) and soluble protein fraction obtained by ultracentrifugation (80,000 *g*, 15 min). For ^14^C-labeling, cells (200 µg chlorophyll, 100 mL) were pre-incubated one hour in an acetate-free Ammonium- or N-free medium and labeled with ^14^C-acetate (5 µCi/mL) for 5 min in the presence of cycloheximide (15 μg/mL).

## 5. Conclusions

In conclusion, we show that ClpP controls the stability of mutant and fully-assembled RuBisCO, and that in ATPase mutant strains it determines the accumulation level and assembly state of α- and β-CF1. These are two major photosynthetic enzymes which assemble in the stroma, where Clp mostly resides. In the case of RuBisCO, the role of ClpP is so crucial that a mutation in *rbcL* makes it impossible even to downregulate the protease. 

Disruption of an assembly pathway by mutations is an extreme situation useful to show the roles of a protease. Another productive approach is the study of nutrient starvation responses, where proteolysis serves both to reorganize the energetic metabolism and to reallocate nutrients stored within abundant proteins. However, a more difficult challenge will be to describe the workings of the intricate network of proteases and substrates during normal assembly in a growing WT cell. This will require much more precise measurements of the biogenetic fluxes, and drugs to selectively inactivate one protease or the other.

## Figures and Tables

**Table 1:** Mutants used in this study.

Mutation	Affected Genes	Mutation	Notes	Reference
*rbcL-G54D (31-4E)* *rbcL-W451Opal (18-5B)*	*rbcL*	G54D W451-Opal	Unstable LSU	Thow and Spreitzer, 1992 Spreitzer et al., 1985
*RBCS-T60*	*RBCS1* (Cre02.g120100)	deletion RBCS1, RBCS2 and STT7 (?)	No SSu, leading to inhibition of LSU translation (CES process)	Khrebtukova and Spreitzer, 1996
*CAL13* *(CAL005_01_13)*	*RBCS2* (Cre02.g120150)	deletion chromosome_2:6,908,477- 6.943.599		Dent et al., 2015
*clpP1-AUU*	*clpP1*	start (AUG)-->AUU	25% accumulation of ClpP complex	Majeran et al., 2000
*Mrl1-5*	*MRL1* *(Cre06.g298300)*	?	No rbcL mRNA accumulation	Johnson, 2011
*Tbc1-F34*	*TBC1 (?)*	?	no PscbC (CP43) translation	Rochaix, et al., 1989
*Tdal-F54*	*TDA1* (Cre08.g358350)	W780-Opal	No *atpA* translation initiation leading to decreased β-CF1 translation (CES process)	Lemaire and Wollman, 1989, Eberhard, et al., 2011
*Mdb1-thm24*	MDB1 (Cre14.g614550)	unknown	No *atp*B mRNA accumulation and inhibition of α-CF1 translation (CES)	Drapier et al., 1992, unpublished
*atpA-FUD16*	*atpA*	I184N and N186Y	aggregation of α-CF1 and β-CF1 in the stroma, forming inclusion bodies	Ketchner et al., 1995

## Supplementary Materials

The following are available online at https://www.mdpi.com/2223-7747/8/7/191/s1, Figure S1: RuBisCO assembly status during S-starvation. The *clpP1-AUU* strain was subjected to S-starvation for 0 (A, C, D) or 24 h (B, E, F) before pulse-chase labeling with ^35^SO_4_^2−^. Soluble fractions were analyzed by 2D-CN-PAGE. In panels A and B, RuBisCO accumulation was monitored by immunoblotting with a holoenzyme antibody. The positions of LSU and SSU are indicated, as well as that of putative LSU assembly intermediates indicated by red stars. The right panels show the LSU region (40–65 kDa) of the autoradiograms. Pulse-labeling was carried out with ^35^SO_4_^2−^ for 7.5 min in the presence of cycloheximide (C and E) and was followed by a 15 min chase (D and F). Similar patterns were observed for the WT strain. NB: In non-starved cells, the 7.5 min duration of the pulse is adequate to visualize chaseable assembly intermediates, but after 24h starvation, the pulse already shows all labeled RuBisCO in the L8S8 form, with no change in the chase. Rather than to an acceleration of RuBisCO biogenesis in starved cells, we attribute this to a faster metabolization of the labeled sulfate, made possible by the induction of sulfate uptake and incorporation systems [55], Figure S2: Selection for double mutants *rbcL-G54D:clpP1-AUU*. (A) Scheme of the expected mutated *clpP1-AUU* genomic region obtained by transformation. Note the silent mutation added in the third codon to create a diagnostic *PvuI* site, underlined. (B) The presence of the construct in the transformants has been verified by PCR with primer couples PA1- cod2 and PA1- AA1 for respectively the WT and aadA-harboring alleles (left panels). Clone 1 is homoplasmic WT and clones 4, 5, 6 are homoplasmic for the aadA cassette, while clones 2 and 3 are heteroplasmic. In the right panel, the PA1- AA1 PCR product has been digested with *PvuI*: Strains 4, 5 and 6 have lost the marker, which can still be observed in the heteroplasmic clones 2 and 3. (C) The accumulation of ClpP1 (low MW form) and LSU was analyzed by western-blot in selected WT homoplasmic (1), heteroplasmic (2 and 3), and homoplasmic recombinant (4, 5 and 6) clones. Western-blot against OEE2 subunit of PSII was used as a loading control, Figure S3: ClpP attenuation partially restores CF1 assembly in the *Fud16 ncc1* background. 1D-CN-PAGE (6–18% gradient) of soluble fractions of WT and ATPase mutant strains, carrying or not the *clpP1-AUU* mutation. Immunoblots revealed with antibodies to α-CF1 (left) or β-CF1 (right). Note that α-CF1 is not detectable in the soluble fraction in *Fud16* because it is entirely within inclusion bodies and is very low in *Fud16 ncc1* because it is degraded. In the ClpP attenuated strain *Fud16clpP1-AUU* and *Fud16 ncc1clpP1-AUU*, a smear probably reflects the formation of aggregates of the mutant α, small enough to remain in the soluble fraction after low-speed centrifugation. Traces of CF1 (red star) are visible on top of the smear, especially in *Fud16 ncc1clpP1-AUU*, detected with both the α- and β-subunit antibodies. In both strains, a putative α3β3 oligomer (orange star) is also detectable. Note that the stabilization of β-subunit in *F54-AUU* background can be observed here again, as seen in Figure 4. The atpC1 mutant [121] carrying an insertion in the ATPC gene for the γ-subunit has been included as control strain totally lacking CF1 subunits, Figure S4: ClpP attenuation partially restores ATPase function in the *Fud16 ncc1* background. A): liquid cultures of (left to right) *Fud16 ncc1*, *Fud16 ncc1clpP1-AUU* and *clpP1-AUU* strains in mineral medium under 35 µmol.m^−2^.s^−1^ constant illumination for several weeks. B): Measurement of photosynthetic electrochromic shift in dark-adapted WT and mutant strains (absorption change at 520 nm), expressed as a percentage of "phase a" value (recorded at 10 µs after the saturating flash, arrow) which represents charge separation in PSI and PSII. After a short rise ("phase b", reflecting the proton-pumping activity of the cytochrome *b*_6_*f* complex), the signal decays, rapidly in the presence of an active ATP synthase, very slowly in its absence. Note the very limited activity observable in the two *Fud16* strains, and the much faster decay rate in *Fud16 ncc1clpP1-AUU*, but not in *Fud16 ncc1*, Figure S5: Attenuation of ClpP does not prevent the loss of photosynthetic electron transfer during S-starvation. Fluorescence parameter ΦPSII (Fm-Fs)/Fm) is plotted as a function of time in S-free medium. The recipient WT strain is compared with two independent transformants carrying *clpP1-AUU* (*clpP1-AUU1* and *-AUU2*) and a control transformant BI carrying the aadA cassette only. The arrow indicates the addition of SO_4_^2−^, initiating recovery, Figure S6: RuBisCO mRNA accumulation is not affected by the clpP1-AUU mutation during S- or N- starvation. The accumulation of *rbcL*, *RBCS1*, and *RBCS2* transcripts was followed by Northern-blot during sulfur starvation (A) and nitrogen starvation (B). *CbLBP* was used as a loading control, Figure S7: CND41 is probably not located in the chloroplast. Start of an alignment of selected CND41 homologs generated using MAFFT and visualized with Bioedit. Sequences with name in red are from Tobacco (CND41 is underlined), in black: Arabidopsis or rice, in orange: lower Streptophytes, in green: *Chlamydomonas*, in violet: other green algae. In CND41, the reported chloroplast transit peptide is italicized and a red arrow points to the mature N-terminal residue. Note conservation of the sequence upstream, and the hydrophobic region of the signal peptide visible in most sequences (bracket). Sequences that TargetP predicts addressed to the secretory pathway are marked by a *.
