# Supplementary material for: Role of ClpP in the Biogenesis and Degradation of RuBisCO and ATP Synthase in Chlamydomonas reinhardtii
Source: Plants (Basel). 2019 Jun 26;8(7):191. doi: 10.3390/plants8070191 (PMC6681370; doi:10.3390/plants8070191)
Supplement: Supplementary file 1 [file plants-08-00191-s001.pdf]

plants

Article

Role of ClpP in the Biogenesis and Degradation of RuBisCO and ATP Synthase in *Chlamydomonas reinhardtii*.

Wojciech Majeran [1,2,*], Katia Wostrikoff [3], Francis-André Wollman [3] and Olivier Vallon [3]

Supplemental Figure S1. RuBisCO assembly status during S-starvation. The *clpP1-AUU* strain was subjected to S-starvation for 0 (A, C, D) or 24 hours (B, E, F) before pulse-chase labeling with $^{35}SO_4^{2-}$. Soluble fractions were analyzed by 2D-CN-PAGE. In panels A and B, RuBisCO accumulation was monitored by immunoblotting with a holoenzyme antibody. The positions of LSU and SSU are indicated, as well as that of putative LSU assembly intermediates indicated by red stars. The right panels show the LSU region (40-65 kDa) of the autoradiograms. Pulse-labeling was carried out with $^{35}SO_4^{2-}$ for 7.5 min in the presence of cycloheximide (C and E) and was followed by a 15 min chase (D and F). Similar patterns were observed for the WT strain. NB: in non-starved cells, the 7.5 min duration of the pulse is adequate to visualize chaseable assembly intermediates, but after 24h starvation, the pulse already shows all labelled RuBisCO in the L8S8 form, with no change in the chase. Rather than to an acceleration of RuBisCO biogenesis in starved cells, we attribute this to a faster metabolization of the labeled sulfate, made possible by the induction of sulfate uptake and incorporation systems [55].

Supplemental Figure S2. **Selection for double mutants *rbcL-G54D:clpP1-AUU*.** (A) Scheme of the expected mutated *clpP1-AUU* genomic region obtained by transformation. Note the silent mutation added in the third codon to create a diagnostic *Pvu*I site, underlined. (B) The presence of the construct in the transformants has been verified by PCR with primer couples PA1- cod2 and PA1- AA1 for respectively the WT and *aadA*-harboring alleles (left panels). Clone 1 is homoplasmic WT and clones 4, 5, 6 are homoplasmic for the aadA cassette, while clones 2 and 3 are heteroplasmic. In the right panel, the PA1- AA1 PCR product has been digested with *Pvu*I: strains 4, 5 and 6 have lost the marker, which can still be observed in the heteroplasmic clones 2 and 3. (C) The accumulation of ClpP1 (low MW form) and LSU was analyzed by western-blot in selected WT homoplasmic (1), heteroplasmic (2 and 3), and homoplasmic recombinant (4, 5 and 6) clones. Western-blot against OEE2 subunit of PSII was used as a loading control.

Supplemental Figure S3. ClpP attenuation partially restores CF1 assembly in the *Fud16 ncc1* background. 1D-CN-PAGE (6-18% gradient) of soluble fractions of WT and ATPase mutant strains, carrying or not the *clpP1-AUU* mutation. Immunoblots revealed with antibodies to -CF1 (left) or -CF1 (right). Note that -CF1 is not detectable in the soluble fraction in *Fud16* because it is entirely within inclusion bodies and is very low in *Fud16 ncc1* because it is degraded. In the ClpP attenuated strain *Fud16clpP1-AUU* and *Fud16 ncc1clpP1-AUU*, a smear probably reflects the formation of aggregates of the mutant , small enough to remain in the soluble fraction after low speed centrifugation. Traces of CF1 (red star) are visible on top of the smear, especially in *Fud16 ncc1clpP1-AUU*, detected with both the - and -subunit antibodies. In both strains, a putative 3 3 oligomer (orange star) is also detectable. Note that the stabilization of -subunit in F54-AUU background can be observed here again, as seen in Figure 4. The *atpC1* mutant [122] carrying an insertion in the *ATPC* gene for the -subunit has been included as control strain totally lacking CF1 subunits.

Supplemental Figure S4. ClpP attenuation partially restores ATPase function in the *Fud16 ncc1* background. A): liquid cultures of (left to right) *Fud16 ncc1*, *Fud16 ncc1clpP1-AUU* and *clpP1-AUU* strains in mineral medium under 35 $\mu E.m^{-2}.s^{-1}$ constant illumination for several weeks. B): Measurement of photosynthetic electrochromic shift in dark-adapted WT and mutant strains (absorption change at 520 nm), expressed as percent of "phase a" value (recorded at 10 µs after the saturating flash, arrow) which represents charge separation in PSI and PSII. After a short rise ("phase b", reflecting the proton-pumping activity of the cytochrome *b6f* complex), the signal decays, rapidly in the presence of an active ATP synthase, very slowly in its absence. Note the very limited activity observable in the two *Fud16* strains, and the much faster decay rate in *Fud16 ncc1clpP1-AUU*, but not in *Fud16 ncc1.*

Supplemental Figure S5. Attenuation of ClpP does not prevent the loss of photosynthetic electron transfer during S-starvation. Fluorescence parameter ΦPSII (Fm-Fs)/Fm) is plotted as a function of time in S-free medium. The recipient WT strain is compared with two independent transformants carrying *clpP1-AUU* (*clpP1-AUU1* and *-AUU2*) and a control transformant BI carrying the aadA cassette only. The arrow indicates addition of SO_4^{2-}, initiating recovery.

Supplemental Figure S6. RuBisCO mRNA accumulation is not affected by the *clpP1-AUU* mutation during S- or N- starvation. The accumulation of *rbcL*, *RBCS1* and *RBCS2* transcripts was

followed by Northern-blot during sulfur starvation (A) and nitrogen starvation (B). CβLBP was used as a loading control.

Supplemental Figure S7. CND41 is probably not located in the chloroplast. Start of an alignment of selected CND41 homologs generated using MAFFT and visualized with Bioedit. Sequences with name in red are from Tobacco (CND41 is underlined); in black: Arabidopsis or rice; in orange: lower Streptophytes; in green: Chlamydomonas; in violet: other green algae. In CND41, the reported chloroplast transit peptide is italicized and a red arrow points to the mature N-terminal residue. Note conservation of the sequence upstream, and the hydrophobic region of the signal peptide visible in most sequences (bracket). Sequences that TargetP predicts addressed to the secretory pathway are marked by a *.

Supplemental Figures.

Supplemental Figure 1

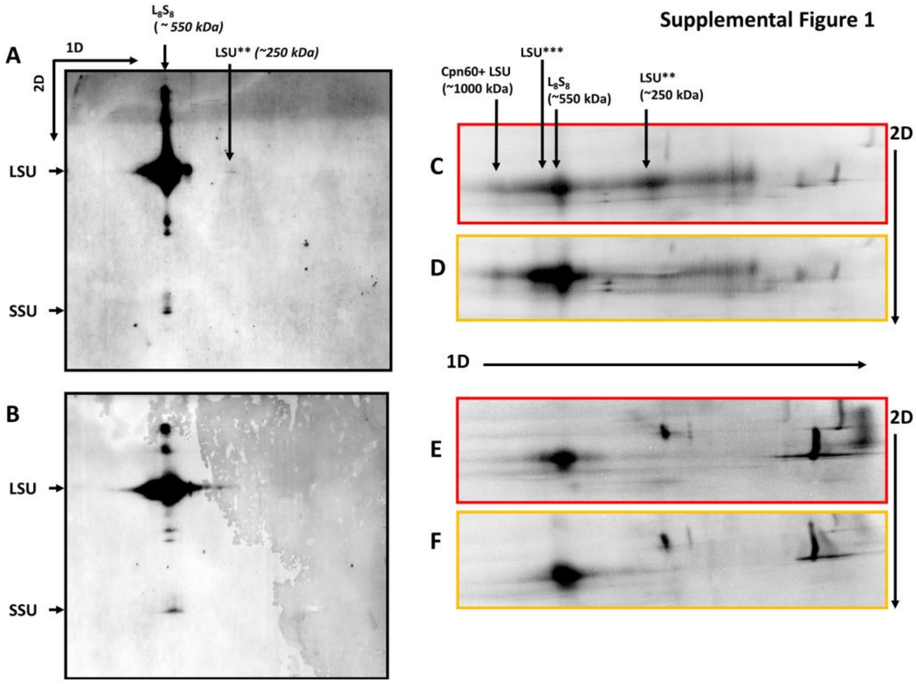

Supplemental Figure 1. RuBisCO assembly status during S-starvation. The *clpP1-AUU* strain was subjected to S-starvation for 0 (A, C, D) or 24 hours (B, E, F) before pulse-chase labeling with $^{35}SO_4^{2-}$. Soluble fractions were analyzed by 2D-CN-PAGE. In panels A and B, RuBisCO accumulation was monitored by immunoblotting with a holoenzyme antibody. The positions of LSU and SSU are indicated, as well as that of putative LSU assembly intermediates indicated by black stars. The right panels show the LSU region (40-65 kDa) of the autoradiograms. Pulse-labeling was carried out with $^{35}SO_4^{2-}$ for 7.5 min in the presence of cycloheximide (C and E) and was followed by a 15 min chase (D and F). Similar patterns were observed for the WT strain. NB: in non-starved cells, the 7.5 min duration of the pulse is adequate to visualize chaseable assembly intermediates, but after 24h starvation, the pulse already shows all labelled RuBisCO in the L8S8 form, with no change in the chase. Rather than to an acceleration of RuBisCO biogenesis in starved cells, we attribute this to a faster metabolization of the labeled sulfate, made possible by the induction of sulfate uptake and incorporation systems (72).

Supplemental Figure 2

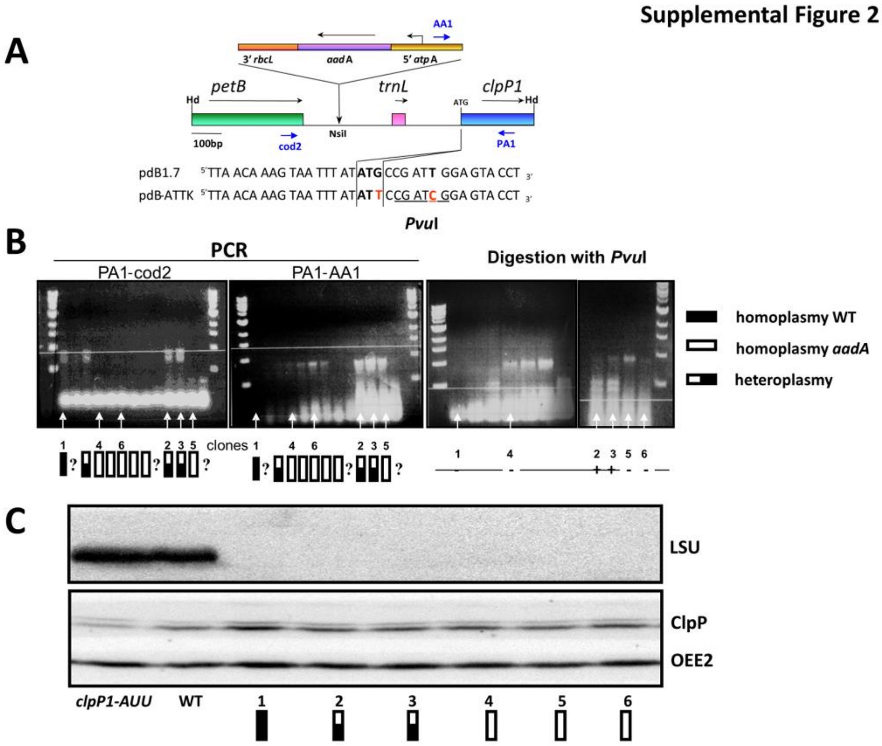

Supplemental Figure 2. **Selection for double mutants *rbcL-G54D:clpP1-AUU*.** (A) Scheme of the expected mutated *clpP1-AUU* genomic region obtained by transformation. Note the silent mutation added in the third codon to create a diagnostic *Pvu*I site, underlined. (B) The presence of the construct in the transformants has been verified by PCR with primer couples PA1- cod2 and PA1- AA1 for respectively the WT and *aadA*-harboring alleles (left panels). Clone 1 is homoplasmic WT and clones 4, 5, 6 are homoplasmic for the aadA cassette, while clones 2 and 3 are heteroplasmic. In the right panel, the PA1- AA1 PCR product has been digested with *Pvu*I: strains 4, 5 and 6 have lost the marker, which can still be observed in the heteroplasmic clones 2 and 3. (C) The accumulation of ClpP1 (low MW form) and LSU was analyzed by western-blot in selected WT homoplasmic (1), heteroplasmic (2 and 3), and homoplasmic recombinant (4, 5 and 6) clones. Western-blot against OEE2 subunit of PSII was used as a loading control.

Supplemental Figure 3

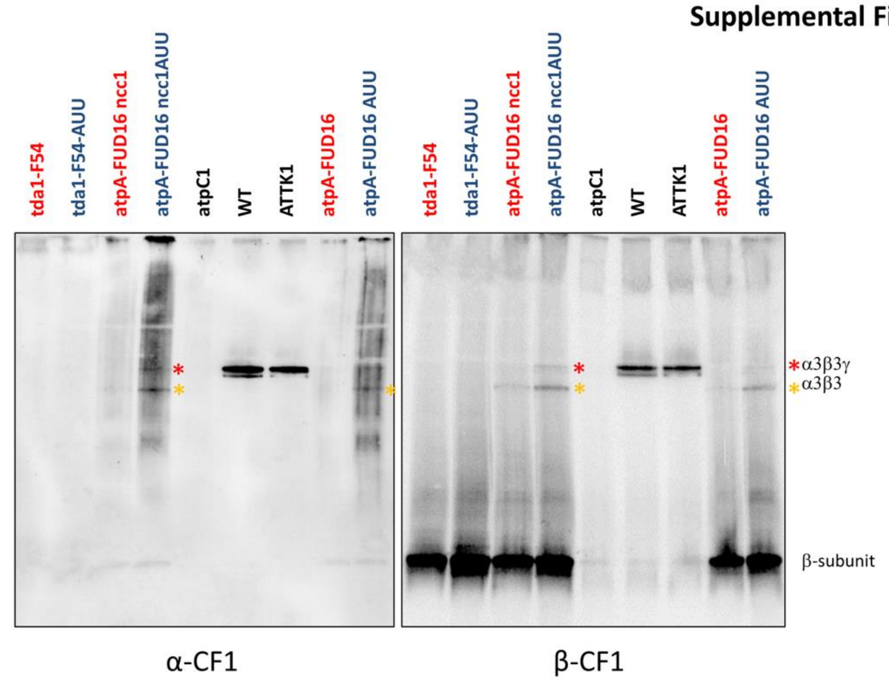

Supplemental Figure 3. ClpP attenuation partially restores CF1 assembly in the *Fud16 ncc1* background. 1D-CN-PAGE (6-18% gradient) of soluble fractions of WT and ATPase mutant strains, carrying or not the *clpP1-AUU* mutation. Immunoblots revealed with antibodies to α-CF1 (left) or β-CF1 (right). Note that α-CF1 is not detectable in the soluble fraction in *atpA-FUD16* because it is entirely within inclusion bodies and is very low in *atpA-FUD16 ncc1* because it is degraded. In the ClpP attenuated strain atpA-*FUD16clpP1-AUU* and *atpA-FUD16 ncc1clpP1-AUU*, a smear probably reflects the formation of aggregates of the mutant α, small enough to remain in the soluble fraction after low speed centrifugation. Traces of CF1 (red star) are visible on top of the smear, especially in *atpA-FUD16 ncc1clpP1-AUU*, detected with both the α- and β-subunit antibodies. In both strains, a putative $\alpha3\beta3$ oligomer (orange star) is also detectable. Note that the stabilization of β-subunit in F54-AUU background can be observed here again, as seen in Figure 4. The *atpC1* mutant (145) carrying an insertion in the *ATPC* gene for the γ-subunit has been included as control strain totally lacking CF1 subunits.

Supplemental Figure 4

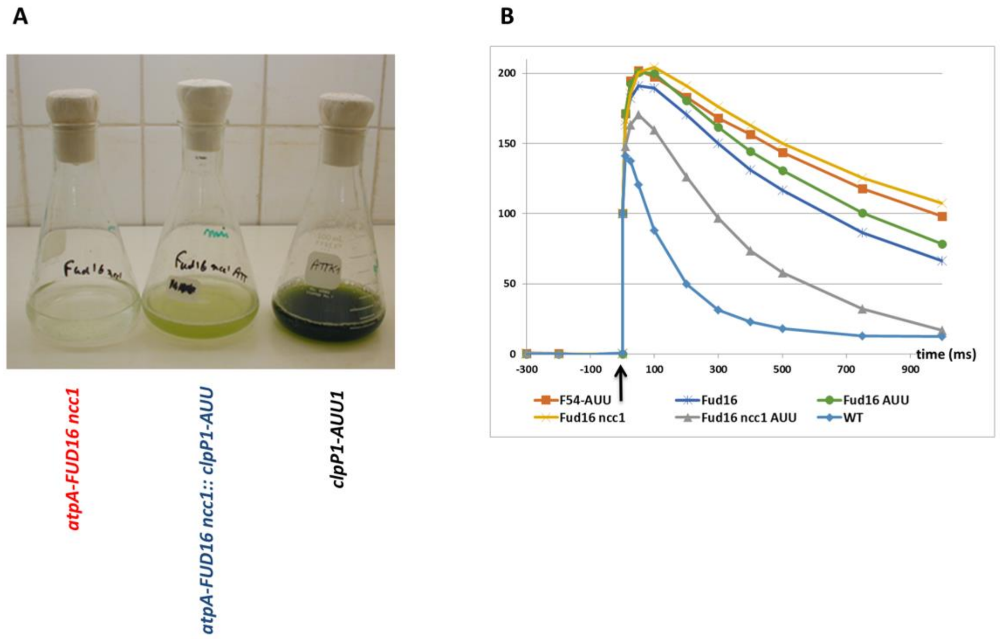

Supplemental Figure 4. ClpP attenuation partially restores ATPase function in the *Fud16 ncc1* background. A): liquid cultures of (left to right) *Fud16 ncc1*, *Fud16 ncc1clpP1-AUU* and *clpP1-AUU* strains in mineral medium under 35 µE.m^{-2}.s^{-1} constant illumination for several weeks. B): Measurement of photosynthetic electrochromic shift in dark-adapted WT and mutant strains (absorption change at 520 nm), expressed as percent of "phase a" value (recorded at 10 µs after the saturating flash, arrow) which represents charge separation in PSI and PSII. After a short rise ("phase b", reflecting the proton-pumping activity of the cytochrome b6f complex), the signal decays, rapidly in the presence of an active ATP synthase, very slowly in its absence. Note the very limited activity observable in the two *Fud16* strains, and the much faster decay rate in *Fud16 ncc1clpP1-AUU*, but not in *Fud16 ncc1*.

Supplemental figure 5

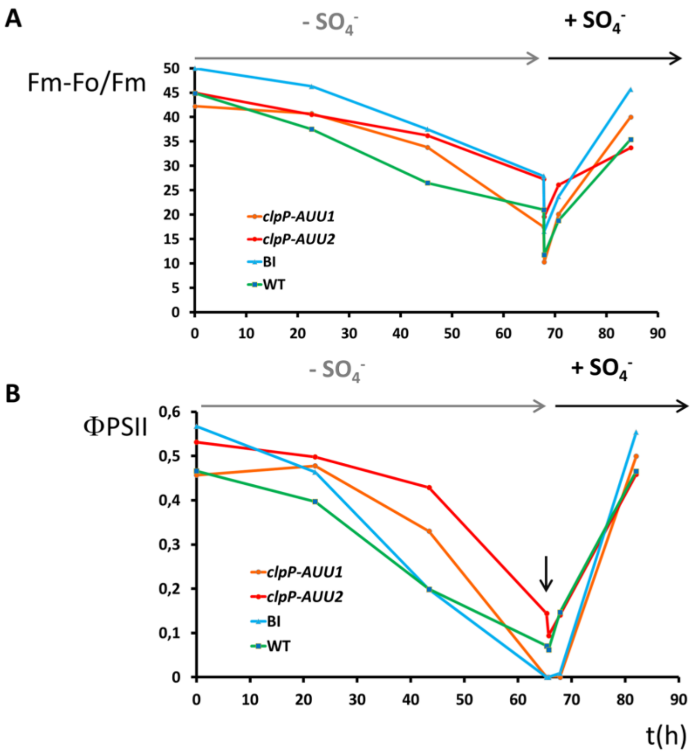

Supplemental Figure 5. Attenuation of ClpP does not prevent the loss of photosynthetic electron transfer during S-starvation. Fluorescence parameter ΦPSII (Fm-Fs)/Fm) is plotted as a function of time in S-free medium. The recipient WT strain is compared with two independent transformants carrying *clpP1-AUU* (*clpP1-AUU1* and *-AUU2*) and a control transformant BI carrying the aadA cassette only. The arrow indicates addition of SO_4^{2-}, initiating recovery.

Supplemental Figure 6

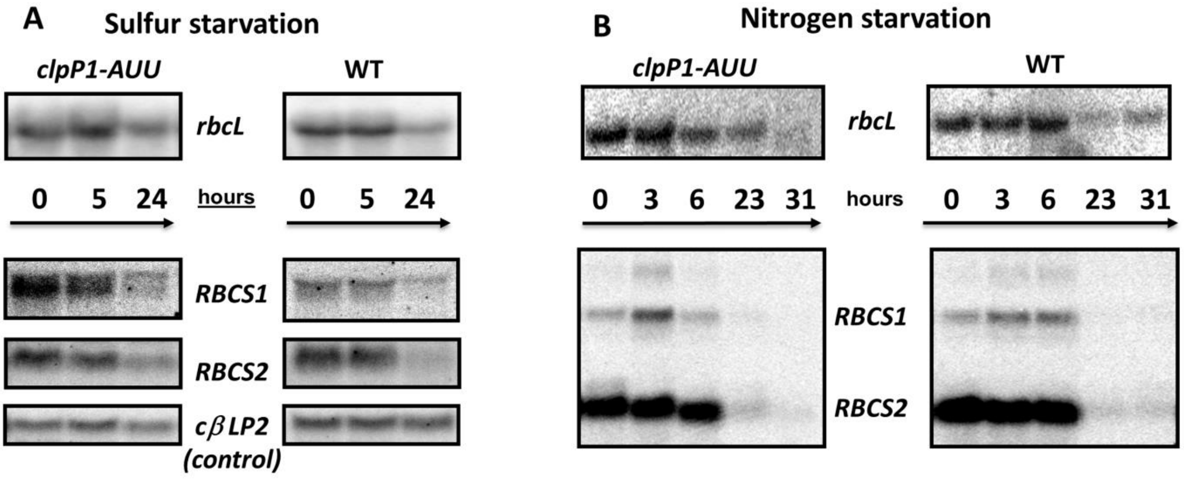

Supplemental Figure 6. RuBisCO mRNA accumulation is not affected by the *clpP1-AUU* mutation during S- or N- starvation. The accumulation of *rbcL*, *RBCS1* and *RBCS2* transcripts was followed by Northern-blot during sulfur starvation (A) and nitrogen starvation (B). CβLBP was used as a loading control.

Supplemental Figure 7

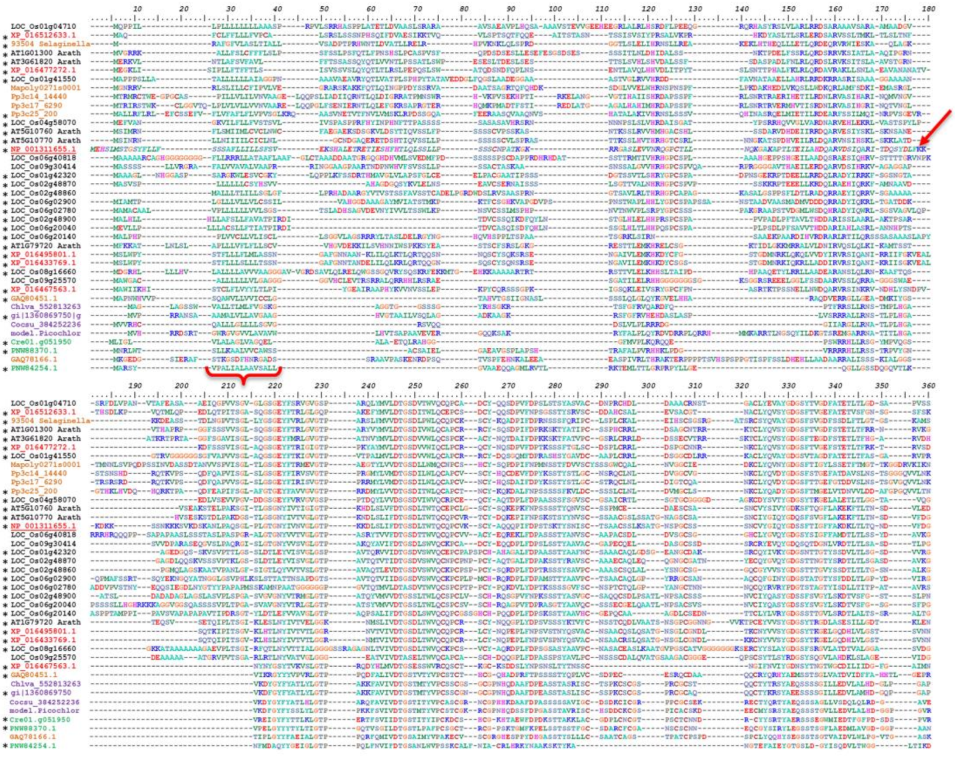

Supplemental Fig 7. CND41 is probably not located in the chloroplast. Start of an alignment of selected CND41 homologs generated using MAFFT and visualized with Bioedit. Sequences with name in red are from Tobacco (CND41 is underlined); in black: Arabidopsis or rice; in orange: lower Streptophytes; in green: Chlamydomonas; in violet: other green algae. In CND41, the reported chloroplast transit peptide is italicized and a red arrow points to the mature N-terminal residue. Note conservation of the sequence upstream, and the hydrophobic region of the signal peptide visible in most sequences (bracket). Sequences that TargetP predicts addressed to the secretory pathway are marked by a *
